# Study on High Activity and Outstanding Stability of Hollow-NiPt@SiO_2_ Core–Shell Structure Catalyst for DRM Reaction

**DOI:** 10.3389/fchem.2020.00220

**Published:** 2020-04-23

**Authors:** Guangying Wang, Yan Liang, Jian Song, Hui Li, Yu Zhao

**Affiliations:** ^1^Anhui Yuanchen Environmental Protection Technology Co., Ltd., Hefei, China; ^2^College of Life and Environmental Sciences, Shanghai Normal University, Shanghai, China

**Keywords:** hollow-NiPt@SiO_2_ core–shell catalyst, renewable energy, dry reforming of methane (CH_4_) (DRM) reaction, NiPt alloy, sintering resistance of SiO_2_

## Abstract

A neoteric hollow-NiPt@SiO_2_ core–shell structure catalyst with 7-nm-sized hollow NiPt alloy nanoparticle (NP) packaged by SiO_2_ shell was prepared by a classic Stober method. Compared with hollow-NiPt/SiO_2_ supported catalyst, the hollow-NiPt@SiO_2_ core–shell catalyst exhibited better activity and thermal stability in dry reforming of methane (CH_4_) (DRM) with CO_2_ reaction, with CH_4_/CO_2_ conversion to 97% and service life to 200 h at 800°C, respectively. In addition, the activity and stability of core–shell catalysts with different nuclei were tested. In contrast to the continuous deactivation of the supported catalyst, all the core–shell catalysts were able to maintain stability for 200 h, and the activity sequence was Hollow-NiPt > NiPt NPs > Pt NPs > Ni NPs. By characterization, we learned that hollow structure had an inner surface and thus had a larger active specific surface area than NP structure. In addition, NiPt NPs had better activity than Ni NPs and Pt NPs because Ni and Pt formed as alloy in NiPt NPs. Therefore, the efficient and thermally stable hollow-NiPt@SiO_2_ core–shell catalyst has a promising application prospect in DRM reaction and can make a considerable contribution to the sustainable use of energy.

## Introduction

Coal and oil are the most important energy consumed in the world; fossil fuels accounted for 85% of global energy consumption in 2018. When fossil fuels are used, a large amount of greenhouse gas-CO_2_ will be produced, accompanied by the generation of polluted flue gas, leading to increasingly serious environmental pollution (Michael et al., 1993; Gurney et al., 2009). On the other hand, coal and oil are non-renewable resources, and the reserves are limited and dwindling. The global fossil-energy revolution has begun, with abundant and cheap natural gas accounting for a growing share of the world's energy consumption. With the breakthrough of shale gas exploitation technology, the application of natural gas has become a worldwide research hotspot in recent years (Wu et al., 2016; Middleton et al., 2017). As the main component of natural gas, there are direct and indirect methods for effective use of methane (CH_4_) (Reddy et al., 2013). Direct transformation of CH_4_ includes oxidation coupling, chlorination coupling, and direct dehydrogenation (Otsuka et al., 1987; Sun and Klabunde, 1999; Zhang et al., 2015). The simple conversion process has potential theoretical advantages. However, due to the difficulty of activation of CH_4_ molecule, direct conversion is usually conducted under harsh conditions of high temperature, high pressure, and high energy consumption. In addition, the complex product composition greatly limits the use of CH_4_. Indirect transformation of CH_4_ includes steam reforming, CO_2_ dry reforming, and partial oxidation, etc. (Iulianelli et al., 2016; Amin et al., 2017; Parola et al., 2018). CH_4_ and CO_2_ can be catalytic converted to syngas (CO and H_2_) by dry reforming of CH_4_ (DRM) reaction. In general, syngas can be converted to liquid fuel by Fischer–Tropsch process or removed from CO via pressure swing adsorption (PSA) to obtain high purity hydrogen (99.999%) which can be used in proton exchange membrane fuel cell (PEMFC) (Miura et al., 2012, 2013; Qiu et al., 2017; Rosli et al., 2017). In recent years, DRM reaction has made significant progress in industrial applications, but large-scale commercial distribution has yet to address the following issues: catalyst thermal stability and carbon resistance (Bian et al., 2016).

The DRM reaction catalysts can be classified into noble metal catalysts and non-noble metal catalysts according to the active metal component. Noble metal catalysts such as Pt, Pd, Rh, and Ru have high activity, high stability, and excellent carbon resistance in DRM reaction (Graf et al., 2007; Özkara-Aydinoglu et al., 2009). Nagaoka et al. (2001a) loaded Pt on ZrO_2_ and Al_2_O_3_, respectively, to study the effect of support on DRM reaction, and results showed that ZrO_2_ had better performance and could run for 500 h without loss of activity. Nagaoka et al. (2001b) studied the high-pressure reaction performance of Ru/TiO_2_ catalyst for DRM reaction and found that the 2%Ru/TiO_2_ catalyst showed excellent carbon resistance at 750°C and 2 MPa pressure. However, the high price and scarce resources of noble metal limit its industrial application prospect, while non-noble metals such as Ni, Co, and Fe also show high initial activity (Nagaoka et al., 2003; Guo et al., 2004; Wang et al., 2011; Djinović et al., 2012). The Ni is relatively cheap and widely used in industrial hydrogen production. However, the most serious issue of Ni-based catalyst is easy to sinter and carbon deposit, leading to deactivation of the catalysts. In order to solve this problem, Ni was modified by using the carbon resistance of noble metal. The work of García-Diéguez et al. (2010) showed that the Pt addition in Ni/Al_2_O_3_ formed the Pt-Ni alloy active site which could promote the reduction of NiO to Ni and inhibit the formation of the inactive site of NiAl_2_O_4_. Liu et al. (2010) showed that adding a small amount of Pd or Pt to Ni/MCM-41 increased the dispersion and reductivity of NiO, although no alloy formation was observed. Nowosielska et al. (2009) found that the DRM activity of Ni-Rh was better than the single metal Ni due to the formation of Ni-Rh alloy active site in the catalyst when studying the modification effect of Rh on Ni/Al_2_O_3_ and Ni/SiO_2_. In addition, Guczi et al. (2010) concluded that Au can also effectively improve the DRM activity and stability of Ni/Al_2_O_3_ as additive.

Due to the rarity of noble metals, how to use them efficiently is the focus. The general method is to reduce the size of the nanoparticles (NPs) (Qiao et al., 2011) or to obtain a special morphology catalyst. Compared with solid NPs, hollow metal nanospheres have become a very effective material to improve the utilization of noble metals due to the advantages of incomparable surface area, lower density, and metal consumption (Kim et al., 2002; Chen et al., 2005; Zhou et al., 2007; Li et al., 2008, 2010). Hollow alloy or bimetallic materials are hot topics in the field of materials research. Chen et al. (2007) synthesized Co-Pt hollow spheres with adjustable composition by an one-step synthesis method; in comparison with Pt NPs, the hollow material showed enhanced electrocatalytic activity toward methanol oxidation. PdCo bimetallic hollow nanospheres synthesized in polyethylene glycol solution was applied to catalysis of the Sonogashira reaction, which displayed obvious advantages of environmentally friendly reaction condition, good catalyst recyclability, simple experimental operation, and high yields (Li et al., 2006). Li et al. (2011) prepared hollow Ni-Pt alloy nanospheres with alterable particle size by an element substitution method, the hollow alloy nanoparticle exhibits higher activity, enhanced selectivity, and better stability than the solid Pt NP on p-chloronitrobenzene hydrogenation reaction.

Since the DRM reaction temperature is required to be very high, usually above 700°C, the sintering resistance ability of the catalyst is also significant. The thermal stability of supported catalyst could usually be improved by increasing the metal–support interaction. However, at such an extremely high DRM reaction temperature, the regular supported catalysts could not completely prevent sintering through such metal–support interaction. It is a reliable strategy to improve the catalyst thermal stability to anchor the metal to the center of the support or between the interlayer by sandwiched structure or core–shell structure. Zhao et al. (2018a) prepared Al_2_O_3_/Ni/Al_2_O_3_ sandwiched catalyst by depositing Al_2_O_3_ thin films on Al_2_O_3_ support to coat Ni NPs through atomic layer deposition (ALD) method, and this catalyst exhibited excellent thermal stability, running stably for over 400 h without activity loss. Core–shell catalysts also maintain outstanding thermal stability in DRM reactions (Zhao et al., 2018b). At present, high-stability core–shell catalysts with various metals (Ag, Au, Pt, Ni, etc.) have been reported except those with hollow-NiPt as the core (Radloff and Halas, 2001; Chen et al., 2013).

**Figure 1:**
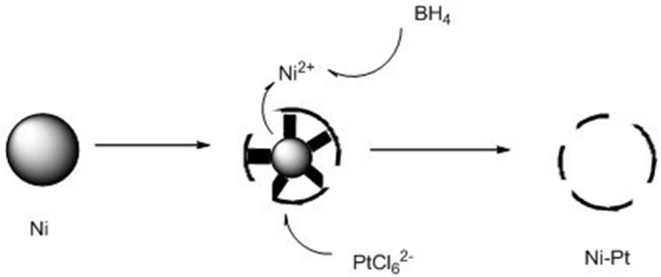
Schematic diagram of synthesis mechanism of hollow-NiPt nanoparticles.

Herein, we reported a novel hollow-NiPt@SiO_2_ core–shell catalyst, which performed excellent activity and operated steadily for 200 h. The hollow-NiPt NPs were prepared by a modified galvanic replacement method as shown in Figure 1. The premise of replacement reaction is oxidation reduction potential. The corresponding standard electrode potentials are: Ni^2+^/Ni, −0.250 eV; ${\text{PtCl}}_{6}^{2-}$/Pt, 0.735 eV. Due to the rapid replacement reaction, platinum atoms nucleate rapidly on the surface of the Ni NP to form smaller particles. Meanwhile, the precipitated Ni^2+^ is rapidly reduced by ${\text{BH}}_{4}^{-}$ and redeposited on the surface of Ni NP. Because the reductant is added drop by drop, the limited ${\text{PtCl}}_{6}^{2-}$ will be dispersed on multiple Ni NPs, and the porous structure on the surface makes the inner Ni metal can be continuously replaced to form the ion diffusion, finally deposited on the outer surface, forming a porous shell structure. Compared with Ni, Pt, and NiPt solid NPs, hollow NPs had better activity, and the stability of core–shell catalysts are better than that of support catalysts. It is clear that the hollow-NiPt catalyst is a suitable catalyst for converting CH_4_ by DRM into syngas, and the SiO_2_ coating provides very reliable thermal stability for the catalyst.

## Experimental Section

### Catalyst Preparation

#### Preparation of Hollow-NiPt@SiO_2_

NiCl_2_·6H_2_O 0.068 g and poly(vinylpyrrolidone) (PVP; MW = 40,000) 0.2 g were dissolved in 400 ml deionized water. The mixed solution was sonicated for 15 min and then purified with N_2_ for another 15 min. NaBH_4_ 80 ml (80.0 mg) solution was added to the above solution drop by drop and stirred at 25°C for 30 min. After pumping 80 ml of K_2_PtCl_6_ (65.6 mg) solution through a peristaltic pump at a speed of 5.0 ml·min^−1^ into the above solution, continue stirring for 30 min to get hollow-NiPt NPs. At room temperature, the resulting 480-ml solution containing hollow-NiPt NPs was mixed with 1,000 ml ethanol solution containing 150 μl tetraethyl orthosilicate (TEOS, 98%), and then 10 ml aqueous ammonia solution was added. After stirring for 6 h, 200 ml toluene was added as settler. The sediment in the bottom was centrifuged and washed with ethanol three times to obtain hollow-NiPt@SiO_2_ NPs.

#### Preparation of Ni@SiO_2_, Pt@SiO_2_, and NiPt@SiO_2_

Ni and Pt NPs with particle sizes of ~7 nm were synthesized by adding 0.77 g nickel(II) acetylacetonate [Ni(acac)_2_, 95%] or 1.17 g platinum(II) acetylacetonate [Pt(acac)_2_, 97%], 1.1 ml tributylphosphate (TBP, 97%), and 2.3 ml trioctylphosphine (TOP, 90%) to 20 ml oleylamine (OAm, 70%) solution. After being treated at 120°C for 2 h in a vacuum oven to remove water and oxygen, the mixture was heated to 220°C and kept heated for 1 h at this temperature. When the solution was cooled to room temperature, the solid product was separated by centrifugation and washed several times with a mixture of ethanol and cyclohexane. The resulting NPs were dissolved in 50 ml cyclohexane.

NiPt NPs with a particle size of about 7 nm were obtained by co-reduction method with organic reductant. Ni(acac)_2_ 0.77 g, Pt(acac)_2_ 1.17 g, and 1,2-hexadecanediol 0.78 g were added to the mixture of 3.2 ml oleic acid (OAc), 4.6 ml TOP, and 10 ml OAm. After the same pretreatment with the removal of water and oxygen as in the previous preparation of Ni and Pt NPs, the solution temperature was controlled to 90°C, and 6 ml OAm [containing 0.79 g borane tributylamine complex (BTB, 97%)] was quickly added and stirred for 1 h. In the same way that Ni and Pt NPs were collected, the resulting NiPt NPs was dissolved in 50 ml of cyclohexane.

The Ni@SiO_2_, Pt@SiO_2_, and NiPt@SiO_2_ core–shell catalysts were all synthesized by a reverse microemulsion method. Organic phase (50 ml cyclohexane), aqueous phase (2.0 ml aqueous ammonia solution), and surfactant (16 ml igepal CO-630) were stirred for 10 min to form a reverse microemulsion. The resulting microemulsion was then mixed with 50 ml cyclohexane containing 3.0 mmol metal NPs. After 30 s of rapid stirring, 2.0 ml tetramethyl orthosilicate (TMOS, 98%) and 2.0 ml octadecyltrimethoxysilane (C_18_TMS, 90%) were added as silicon sources. After stirring for 30 min at room temperature, 10 ml methanol was added as settling agent, and the solid product was separated by centrifugation and washed with ethanol several times.

#### Preparation of Hollow-NiPt/SiO_2_

The supported hollow-NiPt/SiO_2_ catalyst was prepared by impregnation method. The SiO_2_ nanospheres was prepared by the same microemulsion method as above, only without the addition of metal NPs. Hollow-NiPt NPs were dispersed in water as the impregnation liquid.

All catalysts were calcined at 800°C for 4 h prior to activity testing.

### Catalyst Characterization

Metal contents in catalysts were determined by inductively coupled plasma optical emission spectrometry (ICP-OES; Varian VISTA-MPX). We used transmission electron microscopy (TEM; JEOL JEM2100) to observe catalyst microstructure. The catalyst reduction performance was tested by temperature programmed reduction (TPR) on PCA-1200, 0.1 g catalyst was heated to 1,000°C at a heating rate of 5°C/min. The carbon deposition of deactivated catalyst was analyzed by Perkin-Elmer, Pyris Diamond TG/DTA instrument. CO chemisorption was used to analyze the metal dispersion.

### Activity Test

The DRM reaction was performed in a fixed-bed reactor (inner diameter = 10 mm). Before reaction, the catalysts should be reduced under the condition of 10% H_2_/N_2_ (50 ml/min), and then the reduced gas should be switched to N_2_ for 10 min before the reaction gas (CH_4_/CO_2_/N_2_ = 1:1:1) is vented into the reactor. The reaction products were dehydrated and analyzed by an online gas chromatograph (Agilent 7890B GC) with a packed column (TDX-01) and a thermal conductivity detector (TCD). The active data were collected 2 h after the reaction began. The conversions of CH_4_ and CO_2_ were calculated by the following equations:

(1) $$\begin{array}{l} X_{CH_{4}}=\frac{F_{CH_{4},in}-F_{CH_{4},out}}{F_{CH_{4},in}}\times 100 \end{array}$$

(2) $$\begin{array}{l} X_{CO_{2}}=\frac{F_{CO_{2},in}-F_{CO_{2},out}}{F_{CO_{2},in}}\times 100 \end{array}$$

(3) $$\begin{array}{l} F_{i}=F_{total}\times C_{i} \end{array}$$

where X, F, and Ci are conversion, selectivity, gas flow rate, and molar fraction of i in the feed gas or the effluent gas, respectively.

## Results and Discussion

### Structural Characteristics

**Figure 2:**
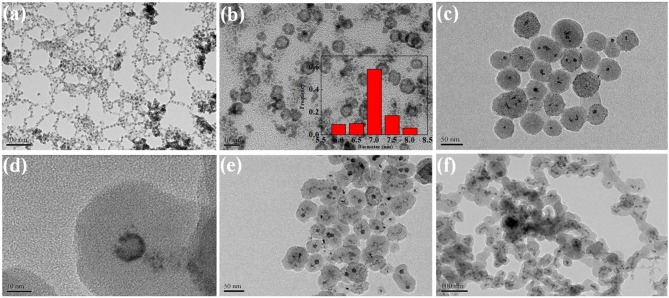
Transmission electron microscopy (TEM) images of **(a)** Hollow-NiPt; **(b)** amplification of Hollow-NiP; **(c)** Hollow-NiPt@SiO_2_ before calcination; **(d)** amplification of Hollow-NiPt@SiO_2_ before calcination; **(e)** Hollow-NiPt@SiO_2_ after calcination and subsequent reduction; **(f)** supported Hollow-NiPt/SiO_2_ after reduction. The attached are the particle size distribution patterns.

**Figure 3:**
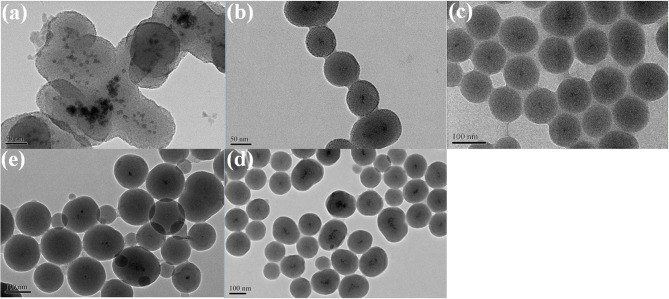
Transmission electron microscopy (TEM) images of hollow-NiPt@SiO_2_ with different tetraethyl orthosilicate (TEOS) addition: **(a)** 100 μl; **(b)** 200 μl; **(c)** 300 μl; **(d)** 500 μl; **(e)** 1,000 μl.

Figure 2 showed the TEM of core–shell and supported structure hollow-NiPt catalyst. It can be seen from the TEM images in Figures 2a,b that the hollow-NiPt NPs are present in the form of hollow nanospheres with a particle size of about 7 nm. In addition to uniform hollow nanospheres, however, there were still some tiny particles, which were generated by direct reduction of minority metal ions not complexed with PVP. In Figures 2c,d, it can be found that the hollow-NiPt@SiO_2_ core–shell structure catalyst is formed after SiO_2_ coating, and SiO_2_ also got the tiny particles in. The total particle size of the core–shell structure is about 50 nm. As shown in Figures 2e,f, after calcination and reduction treatments, the hollow-NiPt NPs in the core–shell structure catalyst remained stable without any agglomeration. On the contrary, the hollow-NiPt NPs in the supported hollow-NiPt/SiO_2_ catalyst had partially aggregated. In addition, we also investigated the effect of TEOS addition on the morphology of the hollow-NiPt@SiO_2_ core–shell catalyst as shown in Figure 3. When 150 μl TEOS was added, the particle size of the core–shell structure catlyst was about 50 nm; when the amount of TEOS was increased to 200, 300, 500, and 1,000 μl, the particle size was about 100, 120, 135, and 140 nm, respectively. On the other hand, when only 100 μl TEOS was added, regular core–shell structure could not be formed. This shows that the TEOS concentration plays a significant role in determining the coating level (Liz-Marzán et al., 1996). However, an excessively thick SiO_2_ shell may lead to difficulty in reactants and products transfer in DRM reaction, resulting in the reaction activity decrease. Therefore, core–shell structure samples with a total particle size of 50 nm were selected for the following activity test to explore whether this thickness could be satisfied to keep hollow-NiPt NPs stable at high temperature.

**Figure 4:**
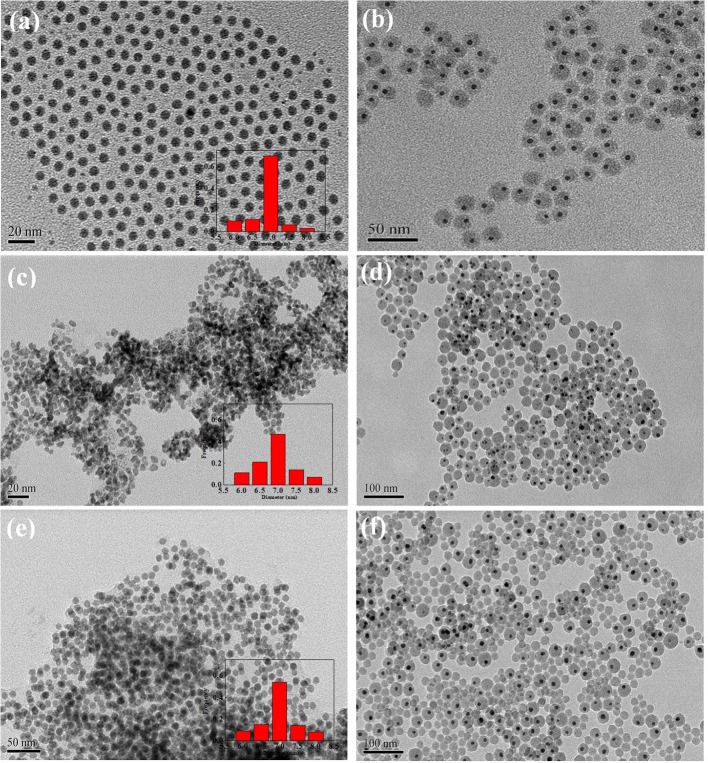
Transmission electron microscopy (TEM) images of **(a)** Ni nanoparticles (NPs); **(b)** Ni@SiO_2_ before calcination; **(c)** Pt NPs; **(d)** Pt@SiO_2_ before calcination; **(e)** NiPt NPs; and **(f)** NiPt@SiO_2_ before calcination. The attached are the particle size distribution patterns.

In order to verify whether the hollow-alloy structure is superior to the solid-single metal structure, we prepared solid Ni, solid Pt, and solid NiPt alloy NPs, respectively. In addition, we also coated them with SiO_2_. For the activity comparison consistent, the particle size of all metal NPs is the same as that of hollow-NiPt, which is controlled at about 7 nm. The thickness of SiO_2_ coating is the same as that of hollow-NiPt@SiO_2_, the total particle size is about 50 nm. As shown in Figure 4, solid Ni@SiO_2_, Pt@SiO_2_, and alloy NiPt@SiO_2_ catalysts were uniform core–shell structures with only one metal NP in one SiO_2_ shell. Besides, the thermal stability of core–shell structures was also expressed in these three catalysts. After calcination, the core–shell structure remained unchanged, and the metal particles were not agglomerated.

**Figure 5:**
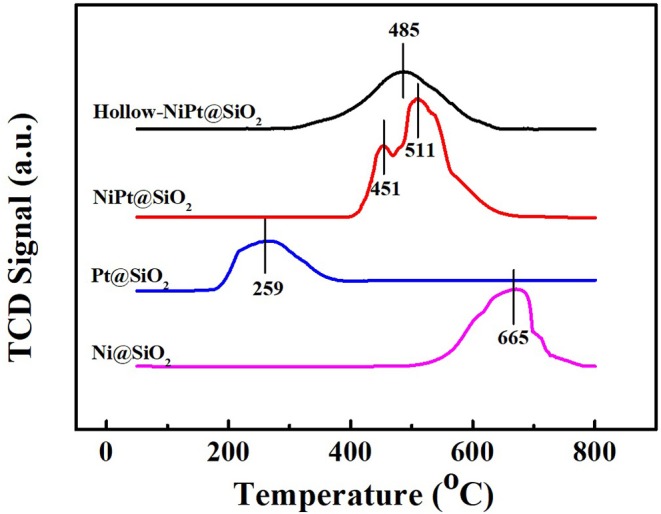
Temperature programmed reduction (TPR) spectras of different catalysts. TCD, thermal conductivity detector.

The TPR profiles in Figure 5 revealed the reducibility of different catalysts. The reduction peaks of Ni and Pt oxides from calcination of Ni and Pt NPs were near 665 and 259°C, respectively. After calcination, the hollow-NiPt catalyst had only a single reduction peak at 485°C, which is between the reduction temperature of Ni and Pt oxides. We could speculate the formation of NiPt alloy in hollow-NiPt catalyst (Yu et al., 1997). It was worth noting that the reduction peak of solid NiPt oxides is in the same region as that of hollow-NiPt catalyst, indicating the formation of alloy in solid NiPt NPs. However, it splits into two peaks, 451 and 511°C, respectively. If solid NiPt NP exists as alloy, the reduction peak must be the same as that of hollow-NiPt NPs. On the contrary, if it exists as a single metal, the reduction peak position must be the same as that of single metal Ni and single metal Pt. Therefore, the splitting of reduction peak demonstrates that solid NiPt NPs are in the intermediate state of single metal and alloy, which manifests that parts of solid NiPt particles are still in the form of single Ni and Pt.

### Catalytic Performance and Stability Test

In order to guarantee the comparability of all test results, the metal content of catalyst in each activity test was constant, which is 0.085 mmol. We can see from Table 1 that the hollow-NiPt@SiO_2_ core–shell catalyst shows the highest initial CH_4_ and CO_2_ conversion compared with other catalysts. In addition, the activity was related to the metal dispersion of the catalyst, because the catalytic reaction takes place on the surface of the active site. This is because all catalytic reactions take place on the catalyst surface, which means that the more active sites exposed to the catalyst surface, the more active sites can participate in the reaction, thus it has higher activity. Nevertheless, with the same NiPt active site, the initial activity of core–shell catalyst was higher than that of the supported catalyst. This may be related to the dispersion state of the reduced hollow NiPi NPs. It can be seen from the TEM in Figure 2f that some NPs agglomerate in the supported catalyst after reduction, which did not appear in the core–shell catalyst (Figure 2e). The aggregation of particles reduced the metal dispersion, thus reducing the utilization of the active sites of the catalyst and ultimately reducing the activity of the catalyst.

**Table 1:** Structural and catalytic parameters of different catalysts^a^.

**Catalysts**	**Hollow-NiPt@SiO_**2**_**	**Ni@SiO_**2**_**	**Pt@SiO_**2**_**	**NiPt@SiO_**2**_**	**Hollow-NiPt/SiO_**2**_**
Metal loading (wt.%)	8.7	13.8	18.9	16.2	9.1
CO adsorption (μmol/g _metal_)	1,597	1,107	1,159	1,143	1,440
Metal dispersio*n* (%)	22.6	15.6	16.3	16.1	20.3
CH_4_ conversio*n* (%)	97.1	87.0	88.7	90.1	94.9
CO_2_ conversio*n* (%)	96.5	87.9	89.3	91.3	94.8

a *Reaction conditions: 800°C, CH_4_:CO_2_:N_2_ = 1:1:1, Gaseous Hourly Space Velocity = 600 ${\text{L·g}}_{\text{M}}^{-1}\cdot$h^−1^*.

Compared with solid core–shell catalysts, hollow-NiPt catalysts had higher metal dispersion and CH_4_/CO_2_ conversions. This is due to the fact that the hollow structure has both internal and external reaction surfaces, which increases the contact point between reactants and active sites and thus improves the availability of noble metal Pt. In solid core–shell catalysts, the activity decreased in the order: NiPt > Pt > Ni, and the activity of Pt was only slightly higher than that of Ni, while the NiPt alloy possessed a large superiority over Pt or Ni. The result showed that the catalytic DRM reaction activity of Ni is comparable to that of Pt at high temperature. The electron transfer between the metals in the alloy catalyst makes the CH_4_/CO_2_ have a higher activation efficiency at the active site and thus have a higher reaction activity, which is also proved by our results.

We were surprised to find that the CH_4_ conversion is a little bit higher than CO_2_ conversion only when the hollow-NiPt catalysts are used, yet it is the opposite in solid catalysts which may be explained by the reverse water gas shift (RWGS) reaction (CO_2_+H_2_→*CO*+*H*_2_O) (Goguet et al., 2004). So we got the conclusion that hollow-NiPt catalysts can effectively reduce the RWGS reaction, thus reducing the generation of by-product H_2_O and improving the selectivity of syngas.

According to the ICP analysis, the metal loadings in the core–shell and supported hollow-NiPt catalysts were determined as 8.7 wt. and 9.1%, which is significantly lower than that in solid catalysts because the hollow NPs have a smaller density and the mass of hollow NPs will be smaller than that of solid NPs with the same size. Similarly, in these solid catalysts, the total metal loading of the catalysts containing the denser Pt was higher.

**Figure 6:**
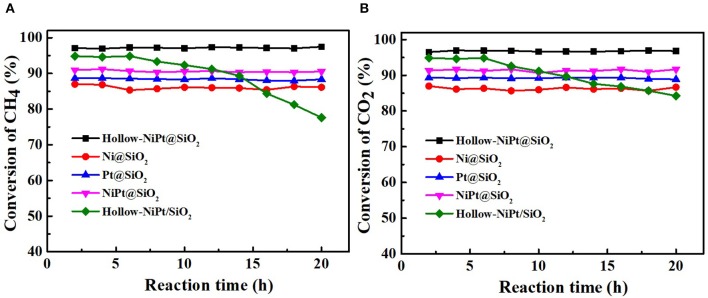
Stability test of all catalysts. **(A)** CH_4_ conversion, **(B)** CO_2_ conversion. Reaction conditions: 800°C, CH_4_:CO_2_:N_2_ = 1:1:1, Gaseous Hourly Space Velocity = 600 ${\text{L·g}}_{\text{M}}^{-1}\cdot$h^−1^.

**Figure 7:**
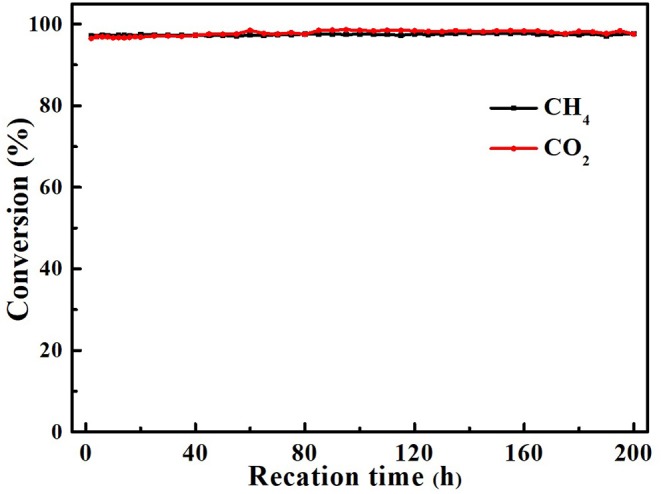
Stability test for Hollow-NiPt@SiO_2_ core–shell catalyst. Reaction conditions: 800°C, CH_4_:CO_2_:N_2_ = 1:1:1, Gaseous Hourly Space Velocity = 600 ${\text{L·g}}_{\text{M}}^{-1}\cdot$h^−1^.

The best way to determine whether the catalyst is suitable for continuous and stable operations in such a high temperature is to use the DRM reaction to investigate its catalytic stability. We tested the activities of all core–shell and supported structure catalysts with Gaseous Hourly Space Velocity of 600 ${\text{L·g}}_{\text{M}}^{-1}\cdot$h^−1^. As shown in Figure 6, within 20 h, all core–shell catalysts maintained the same activity, while the CH_4_ and CO_2_ conversion of supported hollow-NiPt/SiO_2_ decreased from 94.9 to 77.6% and 94.8 to 84.3%, respectively. It can be found that after 20 h reaction, the CO_2_ conversion of hollow-NiPt/SiO_2_ catalyst was significantly higher than that of CH_4_. There may be two reasons here, one is RWGS reaction, the other is the generated carbon deposit, which reacts with CO_2_ to produce CO. The reason why hollow-NiPt@SiO_2_ has better stability than hollow-NiPt/SiO_2_ is that the core–shell structure has better ability to protect metal active sites than the support structure at high temperature, thus reducing the probability of CO disproportionation. The activity of hollow-NiPt@SiO_2_ catalysts was higher than that of hollow-NiPt/SiO_2_ catalysts due to the metal dispersion of catalyst decreases caused by the agglomeration of particles. Figure 7 also reveals the long-term stability test of hollow-NiPt@SiO_2_ catalysts, which can keep stable for 200 h without any CH_4_ and CO_2_ conversion change. This indicates that the SiO_2_-coated hollow-NiPt core–shell catalyst has a considerable industrial application prospect.

**Figure 8:**
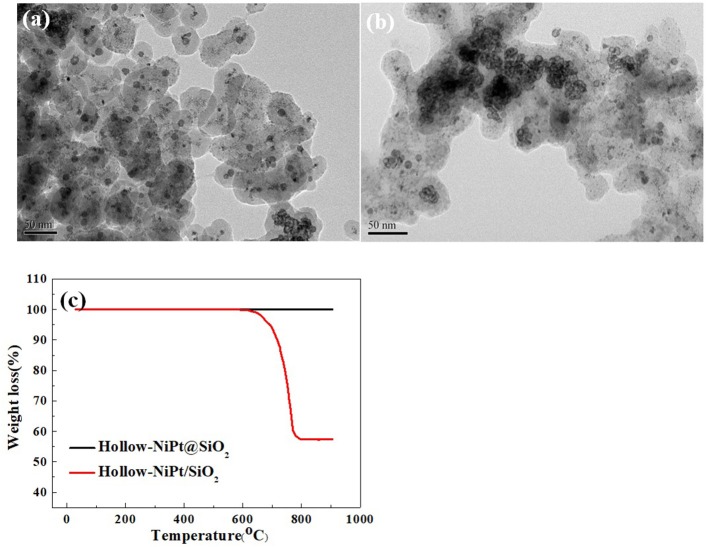
Characterizations of core–shell catalyst and supported catalyst after dry reforming of methane (CH_4_) (DRM) reaction at 800°C for 20 h. **(a)** Transmission electron microscopy (TEM) image of Hollow-NiPt@SiO_2_, **(b)** TEM image of Hollow-NiPt/SiO_2_, **(c)** Thermogravimetric patterns. Reaction conditions: 800°C, CH_4_:CO_2_:N_2_ = 1:1:1, Gaseous Hourly Space Velocity = 600 ${\text{L·g}}_{\text{M}}^{-1}\cdot$h^−1^.

In order to investigate the cause for the divergence of stability between core–shell catalyst and supported catalyst, we characterized the catalysts after 20 h reaction. As shown in Figure 8a, TEM images proclaim that hollow-NiPt@SiO_2_ catalyst still holds its original core–shell structure after reaction, without evident active site aggregation and carbon deposition. Instead, the hollow-NiPt NPs in the supported catalyst in Figure 8b agglomerate seriously, and the SiO_2_ nanospheres change to the carrier without a certain shape. It is aware that water will partially dissociate at high temperature, producing generous H^+^ and OH^−^. OH^−^ will react with SiO_2_, thus destroying the morphology of SiO_2_ nanospheres. After reduction, some hollow-NiPt NPs on the supported catalysts agglomerate, which promotes the generation of by-product H_2_O, thus destroying the SiO_2_ morphology, further sintering NPs, forming a vicious cycle (Keulen et al., 1997).

The thermogravimetric (TG) profile in Figure 8c illustrated that hollow-NiPt@SiO_2_ catalysts produce no carbon deposit after reaction at 800°C for 20 h, but the weight loss of hollow-NiPt/SiO_2_ catalyst was about 20% in the process of heating up. The temperature range of weight loss was about 600–800°C, corresponding to the process of carbon deposit elimination (Yu et al., 2013). There is no obvious carbon crystal lattice in TEM, but this is not the evidence of no carbon deposition because the carbon deposit may exist in the form of amorphous.

## Conclusions

In conclusion, we prepared an original hollow-NiPt@SiO_2_ catalyst for the first time, which took hollow-NiPt as the core and encapsulated it in an inert SiO_2_ shell with a classic TEOS hydrolysis method. A series of core–shell materials with different SiO_2_ thicknesses were obtained by adjusting the amount of TEOS. In the reaction of CH_4_/CO_2_ converted to syngas by DRM reaction, such hollow-NiPt catalysts had considerable activity advantages compared with solid Ni, Pt, or NiPt alloy. The conversion of CH_4_ and CO_2_ can be close to 100%. Furthermore, the catalyst had very good thermal stability and can run stably at 800°C for more than 200 h. By comparing the stability of core–shell catalysts and supported catalysts, we got a conclusion that SiO_2_ can keep hollow-NiPt constancy in DRM reaction at high temperature. At the beginning of the reaction, hollow-NiPt in the supported catalyst began to agglomerate and generate carbon deposit, which showed continuous deactivation. Compared with different solid metal core–shell catalysts, hollow-NiPt@SiO_2_ had pretty good activity due to its double reaction surfaces and alloy effect. On balance, this work provides a strategy for stabilizing the hollow metal material, and the composite material can be used for high temperature reaction.

## Data Availability Statement

All datasets generated for this study are included in the article/supplementary material.

## Author Contributions

Specifically, HL and YZ proposed this topic and design of the project. YL completed the characterization part. GW completed the experimental part. YZ analyzed the results. JS and GW composed the manuscript. All authors participated in the discussions of the results and made important contributions on this work.

## Conflict of Interest

Authors GW, YL, JS and JZ were employed by company Anhui Yuanchen Environmental Protection Technology Co., Ltd. The remaining author declares that the research was conducted in the absence of any commercial or financial relationships that could be construed as a potential conflict of interest.
